# Whole-exome sequencing identified mutational profiles of high-grade colon adenomas

**DOI:** 10.18632/oncotarget.14172

**Published:** 2016-12-25

**Authors:** Sung Hak Lee, Seung Jung Hyun, Tae-Min Kim, Je-Keun Rhee, Hyeon-Chun Park, Min Kim Sung, Sung Soo Kim, Chang An Hyeok, Sug Lee Hyung, Yeun-Jun Chung

**Affiliations:** ^1^ Departments of Hospital Pathology, The Catholic University of Korea, Seoul, Korea; ^2^ Departments of Medical Informatics, The Catholic University of Korea, Seoul, Korea; ^3^ Departments of Microbiology, The Catholic University of Korea, Seoul, Korea; ^4^ Departments of Integrated Research Center for Genome Polymorphism, The Catholic University of Korea, Seoul, Korea; ^5^ Departments of Pathology, The Catholic University of Korea, Seoul, Korea; ^6^ Departments of Cancer Evolution Research Center, The Catholic University of Korea, Seoul, Korea; ^7^ Department of Internal Medicine, The Catholic University of Korea, Seoul, Korea; ^8^ Departments of General Surgery The Catholic University of Korea, Seoul, Korea

**Keywords:** colon, adenoma, high grade adenoma, mutation, whole-exome sequencing

## Abstract

Although gene-to-gene analyses identified genetic alterations such as *APC, KRAS* and *TP53* mutations in colon adenomas, it is largely unknown whether there are any others in them. Mutational profiling of high-grade colon adenoma (HGCA) that just precedes colon carcinoma might identify not only novel adenoma-specific genes but also critical genes for its progression to carcinoma. For this, we performed whole-exome sequencing (WES) of 12 HGCAs and identified 11 non-hypermutated and one hypermutated (*POLE*-mutated) cases. We identified 22 genes including *APC, KRAS, TP53, GNAS, NRAS, SMAD4, ARID2*, and *PIK3CA* with non-silent mutations in the cancer Census Genes. Bi-allelic and mono-allelic *APC* alterations were found in nine and one HGCAs, respectively, while the other two harbored wild-type *APC*. Five HGCAs harbored either mono-allelic (four HGCAs) or bi-allelic (one HGCA) *SMAD4* mutation or 18q loss that had been known as early carcinoma-specific changes. We identified *MTOR*, *ACVR1B*, *GNAQ*, *ATM*, *CNOT1*, *EP300*, *ARID2, RET* and *MAP2K4* mutations for the first time in colon adenomas. Our WES data is largely matched with the earlier ‘adenoma-carcinoma model’ (*APC, KRAS*, *NRAS* and *GNAS* mutations), but there are newly identified *SMAD4*, *MTOR*, *ACVR1B*, *GNAQ*, *ATM*, *CNOT1*, *EP300*, *ARID2, RET* and *MAP2K4* mutations in this study. Our findings provide resource for understanding colon premalignant lesions and for identifying genomic clues for differential diagnosis and therapy options for colon adenomas and carcinomas.

## INTRODUCTION

Colorectal cancer (CRC) is the third most common cancer in males, and the second most common in females worldwide [1]. Although there have been advances in the treatment of CRC and the mortality rate has declined in the past few decades, it remains the fourth most common cause of cancer-related deaths worldwide [2]. The multistep model in the adenoma-to-carcinoma sequence is well recognized in CRC development [3]. According to this model, most CRCs are considered to arise from pre-existing adenomas. Microscopically, conventional colorectal adenomas are classified on the basis of their architectural phenotype as either tubular or villous or tubulovillous type. According to the cellular atypia, they are classified as either low- or high-grade colon adenoma (HGCA) [4]. *APC, KRAS* and *β-catenin* mutations are considered key events in adenoma development while *PIK3CA* and *TP53* mutations are considered key events in the progression to invasive CRC [5–7]. *SMAD4* mutation is rarely detected in colon adenomas but occurs in intramucosal carcinoma and more commonly in invasive carcinomas with metastases [8, 9].

All of these mutations were first identified by conventional gene-to-gene analyses [10, 11]. With advances in sequencing technology (next-generation sequencing, NGS), a comprehensive molecular characterization of CRC has been studied using whole-exome (WES) and/or whole-genome sequencing (WGS) that allows for the interrogation of thousands of variants from multiple genes within a given tumor sample at the same time [12]. Although mutational landscapes of CRC have been reported several times, the genomic data of colon adenomas, especially those generated by high-throughput genome profiling technologies such as WES, is scarce. An earlier study analyzed a case of synchronous colon adenoma and CRC by WES [13]. Another study analyzed 82 nucleotide positions of 14 genes in colon adenomas with various types and grades by targeted sequencing [9]. Due to the small case number or narrow extent of genes analyzed in these reports, they did not reveal a general mutational landscape of colon adenomas. In this study, by using NGS-based WES for 12 HGCAs, we attempted to identify the mutational profiles of HGCA and found several mutations that had not been identified in colon adenomas in previous studies.

## RESULTS

### Whole-exome sequencing and somatic variants

**Figure 1:**
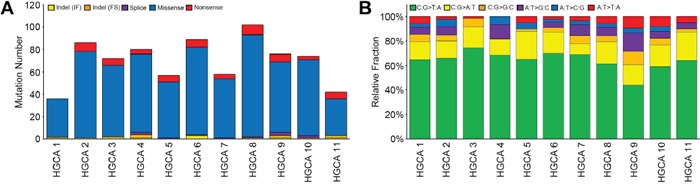
The mutational features of 11 non-hypermutated high-grade colon adenomas **A**. The numbers of non-silent somatic mutations are shown with the five functional categories indicated in the insets. **B**. Non-silent somatic mutations are classified according to both base context and sequence changes. Relative fraction of sequence-based mutation categories (y-axis) for each case is shown. A hypermutated HGCA (HGCA 12) was not shown in this figure.

To obtain the mutational profile of colorectal adenomas, we performed WES for the genome pairs of 12 HGCAs and their matched normals. Mean coverage of the sequencing depth was 98.0X (range: 30.1-149.7X) for normal samples and 115.9X (range: 90.8-150.6X) for HGCA samples (Supplementary Table 1). One HGCA (HGCA12) was found to be a hypermutated adenoma that harbored an exceptionally high incidence of somatic mutations (883 non-synonymous single nucleotide variants (SNVs) and 12 indels). It had a somatic mutation (p.E491K) in *polymerase ε* (*POLE*) gene that encodes the catalytic and proofreading subunits of the DNA polymerase ε [12]. Excluding this hypermutated HGCA, a total of 772 somatic non-silent mutations (752 non-synonymous SNVs and 20 indels) (average: 70.2, range: 36-102) were identified from the other 11 HGCAs (Figure 1A, Supplementary Table 2 and Supplementary Table 3). The C: G->T: A transitions were the most significant changes in the HGCA samples (Figure 1B and Supplementary Table 2), which was consistent with a previous report that analyzed a colon adenoma [13]. Based on the notion that DNA methylation frequently occurs within the context of CpG sites, the enrichment of C: G->T: A transitions in 5′-CpG-3′ dinucleotides might be related to extensive DNA methylation at 5′-CpG-3′ sites in CRC tumorigenesis [14, 15].

**Figure 2:**
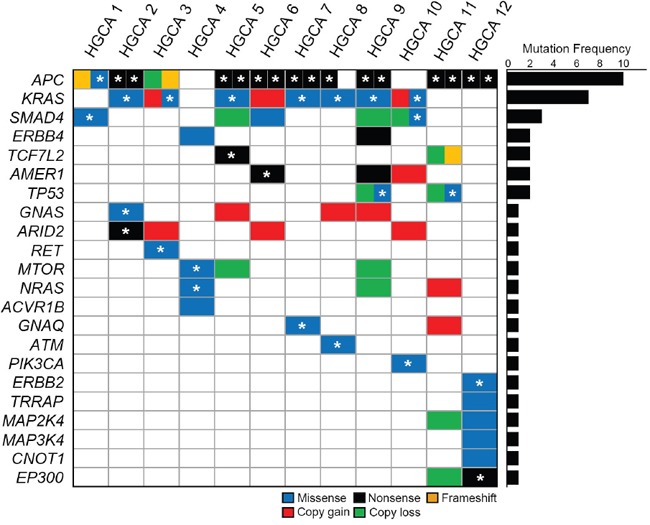
Somatic mutations and copy number alterations of tumor-related genes in high-grade colon adenomas Twenty-two candidate driver genes are mutated in the adenomas. Block colors represent the functional categories of mutation and copy number alteration. Asterisks represent the somatic mutations that overlap the COSMIC database at variant level.

To address whether the mutations found in our study were causally implicated in tumor development, we looked up the cancer Gene Census [16] and the Cancer Driver Database [17] with the CHASM [18] analysis. In this study, mutations that were significantly predicted as drivers (FDR < 0.2) in the CHASM analysis and overlapped the driver databases (the cancer Gene Census or the Cancer Driver Database) were considered potential driver mutations. Under this criterion, 17 genes were considered the candidate driver genes (*APC, KRAS, SMAD4, GNAS, RET, NRAS, MTOR, ACVR1B, GNAQ, ATM, TP53, PIK3CA, ERBB2, TRRAP, MAP2K4, MAP3K4* and *CNOT1*). In addition, six genes with truncating mutations overlapped the driver databases were also included (*APC*, *ARID2*, *ERBB4*, *TCF7L2*, *AMER1* and *EP300*). Overall, we defined 22 genes as candidate drivers (Table 1 and Figure 2). Mutations identified with WES were validated by either digital-polymerase chain reaction (PCR) or Sanger sequencing (Supplementary Figure 1). Nine genes with mutations identified in this study overlapped the CRC top 20 genes in the COSMIC database ( http://cancer.sanger.ac.uk/cosmic) (*TP53, TCF7L2, PIK3CA, AMER1*, *APC, KRAS, SMAD4, GNAS* and *NRAS*). Of note, most of the driver genes (16/22 genes; *APC, KRAS, SMAD4, MTOR, TCF7L2, AMER1, TP53, GNAS, NRAS, ARID2, GNAQ, ATM, PIK3CA, ERBB2, EP300* and *RET*) were identified to harbor the same mutations at variant levels in the COSMIC mutation database as well. Among all of the mutated genes, 13 genes were recurrently mutated in more than three HGCA cases: *APC* (n=10), *KRAS* (n=7), *TTN* (n=7), *OBSCN* (n=5), *CSMD1* (n=4), *MXRA5* (n=4), *SMAD4* (n=3), *FAT2* (n=3), *CELSR2* (n=3), *CSMD2* (n=3), *MAP2* (n=3), *ANK2* (n=3) and *HMCN1* (n=3) (Supplementary Table 3). As these genes besides *APC, KRAS* and *SMAD4* are listed in neither of the cancer Census Genes nor the COSMIC CRC genes, they might be passenger mutations or HGCA-specific mutations that might not play an important role in HGCA progression to CRC.

**Table 1:** Non-silent somatic mutations identified in the cancer Census Genes

Gene	Case	Exonic function	cDNA Change	Amino acid
*APC*	HGCA 1	frameshift	c.4385_4386delAG	p.K1462fs
	HGCA 1	missense	c.1958G>A	p.R653K
	HGCA 2	nonsense	c.1660C>T	p.R554X
	HGCA 2	nonsense	c.4189G>T	p.E1397X
	HGCA 3	frameshift	c.4385_4386delAG	p.K1462fs
	HGCA 5	nonsense	c.847C>T	p.R283X
	HGCA 5	nonsense	c.4067C>A	p.S1356X
	HGCA 6	nonsense	c.4348C>T	p.R1450X
	HGCA 6	nonsense	c.3340C>T	p.R1114X
	HGCA 7	nonsense	c.4222G>T	p.E1408X
	HGCA 7	nonsense	c.3340C>T	p.R1114X
	HGCA 8	nonsense	c.1690C>T	p.R564X
	HGCA 9	nonsense	c.3093T>A	p.Y1031X
	HGCA 9	nonsense	c.4348C>T	p.R1450X
	HGCA 11	nonsense	c.3871C>T	p.Q1291X
	HGCA 11	nonsense	c.2626C>T	p.R876X
	HGCA 12	nonsense	c.694C>T	p.R232X
	HGCA12	nonsense	c.2413C>T	p.R805X
*KRAS*	HGCA 2	missense	c.35G>T	p.G12V
	HGCA 3	missense	c.35G>A	p.G12D
	HGCA 5	missense	c.35G>T	p.G12V
	HGCA 7	missense	c.35G>A	p.G12D
	HGCA 8	missense	c.34G>T	p.G12C
	HGCA 9	missense	c.34G>A	p.G12S
	HGCA 10	missense	c.38G>A	p.G13D
*SMAD4*	HGCA 1	missense	c.1082G>A	p.R361H
	HGCA 6	missense	c.1212C>A	p.D404E
	HGCA 10	missense	c.1156G>A	p.G386S
*ERBB4*	HGCA 4	missense	c.1753G>T	p.D585Y
	HGCA 9	nonsense	c.2224G>T	p.E742X
*TCF7L2*	HGCA 5	nonsense	c.70G>T	p.E24X
	HGCA 11	frameshift	c.463delA	p.K155fs
*AMER1*	HGCA 6	nonsense	c.1072C>T	p.R358X
	HGCA 9	nonsense	c.1240C>T	p.Q414X
*TP53*	HGCA 9	missense	c.524G>A	p.R175H
	HGCA 11	missense	c.747G>C	p.R249S
*GNAS*	HGCA 2	missense	c.601C>T	p.R201C
*ARID2*	HGCA 2	nonsense	c.3817C>T	p.R1273X
*RET*	HGCA 3	missense	c.1267G>A	p.G423R
*MTOR*	HGCA 4	missense	c.6644C>T	p.S2215F
*NRAS*	HGCA 4	missense	c.183A>C	p.Q61H
*ACVR1B*	HGCA 4	missense	c.101G>A	p.C34Y
*GNAQ*	HGCA 7	missense	c.286A>T	p.T96S
*ATM*	HGCA 8	missense	c.1010G>A	p.R337H
*PIK3CA*	HGCA 10	missense	c.3140A>G	p.H1047R
*ERBB2*	HGCA 12	missense	c.2524G>A	p.V842I
*TRRAP*	HGCA 12	missense	c.10751T>A	p.V3584E
*MAP2K4*	HGCA 12	missense	c.881G>A	p.G294E
*MAP3K4*	HGCA 12	missense	c.4577C>T	p.A1526V
*CNOT1*	HGCA 12	missense	c.7048G>A	p.E2350K
*EP300*	HGCA12	nonsense	c.3934C>T	p.R1312X

HGCA: high-grade colon adenoma

We compared our mutation data with those of two previous studies that had analyzed colon adenomas by either WES or targeted NGS [9, 13]. Of the mutated genes in our study, *APC, KRAS* and *NRAS* mutations were identified in the previous studies, but *SMAD4* mutations were not. Not only the *APC* and *KRAS* mutations, but also *TP53* (12.8% prevalence), *NRAS* (1.9%), *AMER1* (6.9%), *TCF7L2* (6.9%), *MAP3K4* (3.4%), *TRRAP* (3.4%), *ERBB2* (3.4%), *ERBB4* (3.4%), *TRRAP* (3.4%), *PIK3CA* (1.3%) and *GNAS* (12.1%) mutations were catalogued in the COSMIC database of colon adenoma. In the COSMIC database, 0.8% (2/246) of *SMAD4* mutations have been reported in colon adenomas. The COSMIC database, however, reported no *ATM, EP300, MTOR, CNOT1, ARID2, RET, MAP2K4, ACVR1B* and *GNAQ* mutations in colon adenomas. Their mutations were reported 8.9%, 6.0%, 5.4%, 5.1%, 4.9%, 3.4%, 2.4%, 1.7% and 0.9% in CRCs, respectively.

Next, we analyzed distribution of the tumor-related gene mutations in the 12 HGCAs. Non-hypermutated and hypermutated HGCA per case harbored 2-5 mutations and 7 mutations, respectively (Figure 2). *APC* gene was somatically mutated in all HGCAs except two cases, which harbored *NRAS* and *MTOR* mutations (HGCA4) and *KRAS*, *SMAD4* and *PIK3CA* mutations (HGCA10).

Of the ten HGCA cases with *APC* mutations, eight harbored bi-allelic *APC* mutations, while two cases (HGCA3 and HGCA8) harbored mono-allelic *APC* mutations (Figure 2). All of the *KRAS* mutations were detected either in the amino acid residue p.G12 or p.G13 that is a mutational hotspot [19, 20]. Of the *SMAD4* mutations (p.R361H, p.G386S and p.D404E), p.R361H and p.G386S have been found in many cancers, including colon, stomach and esophageal cancers [21], while p.D404E is a novel mutation that has not been reported. Missense mutations of *TP53* (p.R175H and p.R249S) in this study have been reported in many cancers including colon adenoma [22] and p.R175H is one of the mutational hotspots in *TP53* (the COSMIC database). Two HGCAs harbored nonsense mutations (p.R358X and p.Q414X) of *AMER1*, which encodes an X-linked negative regulator of WNT signaling [23]. Mutations of *AMER1, TCF7L* and *MTOR* have been reported in CRC [12, 24]. One case (HGCA4) harbored an *NRAS* hotspot mutation p.Q61H, which has been reported in many cancers, including thyroid, lung, skin and CRC [25]. *PIK3CA* mutation identified in *APC* wild-type case (HGCA10) was the hotspot mutation p.H1047R that is common in CRC as well [12]. *ATM* p.R337H mutation was the hotspot site and common in colon and liver cancers [26]. *GNAQ* p.T96S mutation has been reported in skin and prostate cancers [27, 28]. *GNAS* mutation identified in this study was the hotspot mutation p.R201C that is common in low-grade appendiceal mucinous neoplasm and pancreatic intraductal papillary mucinous neoplasm [29, 30]. Nonsense mutation of *ARID2* p.R1273X in our study has been reported in esophageal and head/neck cancers [31, 32]. *RET* p.G423R mutation has been reported in hairy cell leukemias [33]. *ERBB2* p.V842I and *EP300* p.R1312X mutations have been reported in colon and ovary cancers, respectively [12, 34].

### Copy number alterations

We analyzed somatic copy number alterations based on the read depth difference in the exome sequencing data between HGCA and matched normal tissues. Of the tumor-related gene mutations detected, 13 genes were accompanied by copy number alterations as well (Figure 2 and Supplementary Table 4). Copy number gains were found in six genes (*KRAS, AMER1, GNAS, ARID2, NRAS* and *GNAQ*) and copy number losses in eight genes (*APC*, *SMAD4*, *TCF7L2*, *TP53*, *MTOR*, *NRAS*, *MAP2K4* and *EP300*). Of note, the case with mono-allelic *APC* mutation (HGCA3) exhibited copy loss as well, suggesting a bi-allelic alteration (mutation + copy loss). Likewise, two cases with mono-allelic *TP53* mutation (HGCA9 and HGCA11) also exhibited copy loss in the locus. As for *KRAS*, in total, eight of twelve HGCAs (67%) exhibited mutation and/or copy gain. Similarly, *SMAD4* gene showed mutation or copy loss in five of twelve HGCAs (42%).

### Pathway analysis of mutated genes in the HGCA

The mutated genes identified in HGCAs were annotated through the Kyoto Encyclopedia of Genes and Genomes (KEGG) pathway analysis using DAVID tools [35] and found that mutated genes in the HGCA were significantly associated with tumorigenesis-related gene functions (Supplementary Table 5).

## DISCUSSION

Comprehensive analysis of genomic profiles is crucial for understanding tumorigenic mechanism in cancer development. Compared to CRC, mutational profiles of HGCA, a premalignant lesion that precedes invasive CRC, are not well studied. The aim of this study was twofold. First, we attempted to disclose the genomic profiles of HGCAs to find the mutational abundance compared to that of CRC especially with respect to the driver mutations. The next aim was to find cancer-related gene mutations that emerge from the HGCA stage rather than invasive CRC stage. Consistent with the previous report, total numbers and spectrum of somatic mutations identified in the HGCAs were not significantly different from those in CRCs [12]. We found 22 tumor-related gene mutations (*APC, KRAS, SMAD4, ERBB4, TCF7L2, AMER1, TP53, GNAS, ARID2, RET, MTOR, NRAS, ACVR1B, GNAQ, ATM, PIK3CA, ERBB2, TRRAP, MAP2K4, MAP3K4, CNOT1* and *EP300*) from 12 HGCAs that harbored 2-7 of these mutations per case. Presence of *APC, KRAS, NRAS*, and *GNAS* mutations in HGCA well matched ‘classical adenoma-carcinoma model’ [20, 36]. As for *TP53* and *PIK3CA*, their mutations are known to occur mainly in the late stage of the CRC model (invasive carcinoma) [20]. However, an earlier study [36] reported that *TP53* mutations were fairly common in colon adenomas albeit less than CRC. *PIK3CA* mutations are rare in in colon adenoma, but often occur in advanced adenomas greater than 5 cm in diameter [37]. In our study, the HGCA10 with *PIK3CA* mutation was found in a large adenoma (3.7 cm in diameter) with a synchronous carcinoma component, suggesting that the HGCA case may be a full-grown late-stage one. *SMAD4* mutation that had seldom been reported in HGCAs was identified in our study. Also, previously unreported mutations in colon adenomas (*MTOR*, *ACVR1B*, *GNAQ*, *ATM*, *CNOT1*, *EP300*, *ARID2, RET* and *MAP2K4*) were newly discovered in this study.

Earlier studies showed that *SMAD4* mutation followed *APC* mutation and precedes *TP53* mutation in CRC development [20, 38]. Our data on *SMAD4* mutation in HGCAs is in agreement with the gene sequence (*APC-SMAD4-TP53* mutations). However, it is not in agreement with the previous notion that *SMAD4* mutations rarely occurred either in adenoma or intramucosal carcinoma, but was common in advanced CRCs [8, 38]. *SMAD4* somatic mutations have been reported in 0.8% of colon adenomas (the COSMIC database), in 0% of low- and high-grade adenomas and 0% of adenomas and intramucosal carcinomas [8, 9], indicating *SMAD4* mutation is a rare event in early CRC tumorigenesis. The patterns of *SMAD4* alteration in our HGCAs are somewhat different from those described in advanced CRCs where *SMAD4* mutations were usually accompanied by allelic loss of 18q where *SMAD* gene resides [8]. Of the five *SMAD4* alterations four (HGCA1, HGCA5, HGCA6 and HGCA9) were either somatic mutation or 18q loss, indicating they were mono-allelic, while one (HGCA10) harbored both 18q loss and somatic mutation. Size of HGAC10 (3 cm in diameter) and with a synchronous CRC, suggesting that it may be a late-stage adenoma. Together, our data suggest that mono-allelic inactivation of *SMAD4* either by mutation or copy loss may occur in HGCAs and that it may require additional hit during or after the progression to invasive or metastatic CRC.

The mutations (*MTOR*, *ACVR1B*, *GNAQ*, *ATM*, *CNOT1*, *EP300*, *ARID2, RET* and *MAP2K4*) newly detected in HGCAs in the present study have been reported in CRCs at low incidences (0.9%-8.9%) as well as other cancers [31–33]. The COSMIC database, however, shows that there is no mutation for these genes in 29 colon adenomas. Together with this, our data indicate that these gene mutations albeit low incidences may occur in adenomas, at least in HGCAs, and might possibly contribute to early CRC development.

Somatic mutations of *POLE* in the exonuclease domain are found in CRC and endometrial cancers that show association with hypermutability [39]. *POLE* somatic mutations in these cancers were recurrent in seven amino acids (p.P286R/H/L, p.S297F/Y, p.V411L, p.P436R, p.M444K, p.A456P and p.S459F), but the *POLE* p.E491K mutation in our result has not been reported. Association of germline *POLE* mutation with familial adenoma development suggests that *POLE* mutation might occur as an early event in sporadic tumors [40], but there has been no such data that support the hypothesis. In this study, we show data supporting that loss of proofreading activity by *POLE* mutation could play a role in early CRC development.

In summary, our study for the first time attempted to disclose mutational profiles of HGCAs by WES that had not been analyzed before except a study for one case. Our data is largely in agreement with the earlier ‘colorectal adenoma-carcinoma model’ that was made by gene-to-gene approaches [10, 11]. Our HGCA mutation list includes not only those in this classical model (*APC* and *KRAS*) but also those already known as adenoma genes (*NRAS* and *GNAS*). In addition, we newly identified *SMAD4*, *MTOR*, *ACVR1B*, *GNAQ*, *ATM*, *CNOT1*, *EP300*, *ARID2, RET* and *MAP2K4* in HGCAs. Our data are based on the analysis of twelve HGCA genomes. The small size of the study set is due to the difficulties in obtaining histologically defined fresh HGCA tissues in a single institution since in most of the cases they are used up for diagnostic purpose. Further investigation in a larger cohort of HGCA may reveal valuable information to confirm our data, e.g., *SMAD4* mutation, 18q loss and other newly discovered mutations. Also, such an approach would reveal additional information, e.g., discovery of potential additional driver mutations in HGCAs. In addition, meta-analysis of multiple cases with diverse ethnic backgrounds may be required to investigate whether such mutational contexts are population-specific. Our findings may provide a useful resource for understanding this premalignant disease and identifying genomic clues for differential diagnosis and therapy options for colon adenoma and carcinoma.

## MATERIALS AND METHODS

### Tumor specimen and DNA extraction

Fresh frozen normal and HGCA tissues from 12 Korean patients were obtained from the tissue banks of Seoul Saint Mary Hospital (Seoul, Korea), Guro Hospital of Korea University (Seoul, Korea) and Busan National University Hospital (Busan, Korea). All of the cases were sporadic, without any family history of CRC. Clinicopathologic features of the 12 HGCA patients are summarized in Table 2. We defined HGCA according to the World Health Organization [41] where is defined by architectural complexity and cytologic features including extent of nuclear stratification and severity of abnormal nuclear morphology. All of the samples from tumor and normal areas were frozen, cut, and stained with hematoxylin and eosin. A pathologist identified that HGCA lesions were in the samples (Supplementary Figure 2). All cases were tubulovillous adenomas (Table 2). We observed high grade dysplasia in both tubular and villous components in all cases. HGCA areas with rich tumor cell population (at least 70%) were selected and sliced from the frozen tissues with clean blades, and were subsequently used in the study. We also examined the histology by FFPE that showed definite HGCA. After the slicing, we examined again the histology of remnant frozen tissues under microscope and found no changes in the histologic diagnoses of HGCA. We used normal tissues as controls belonged to the same patients. For genomic DNA extraction, we used the DNeasy Blood & Tissue Kit (Qiagen, Hilden, Germany), according to the manufacturer's instructions. The study protocol was reviewed and approved by the Institutional Review Board of the Catholic University of Korea, College of Medicine (MC14SISI0033).

**Table 2:** Clinicopathologic parameters of 12 high-grade colon adenoma patients

Case	Age/Sex	Size in diameter (cm)	Location in colon	Diagnosis	Accompanied malignant lesion	Tumor cell content (%)
HGCA 1	51/F	3.8	descending	Tubulovillous adenoma with high grade dysplasia	No	>70%
HGCA 2	71/M	8.0	descending	Tubulovillous adenoma with high grade dysplasia	No	>70%
HGCA 3	71/F	3.2	cecum	Tubulovillous adenoma with high grade dysplasia	No	>70%
HGCA 4	67/M	4.0	sigmoid	Tubulovillous adenoma with high grade dysplasia	No	>70%
HGCA 5	62/M	4.0	rectum	Tubulovillous adenoma with high grade dysplasia	Intraepithelial adenocarcinoma	>70%
HGCA 6	58/M	2.5	sigmoid	Tubulovillous adenoma with high grade dysplasia	Intraepithelial adenocarcinoma	>70%
HGCA 7	54/F	3.0	ascending	Tubulovillous adenoma with high grade dysplasia	No	>70%
HGCA 8	77/M	1.0	Descending	Tubulovillous adenoma with high grade dysplasia	No	>70%
HGCA 9	60/M	1.5	Sigmoid	Tubulovillous adenoma with high grade dysplasia	Invasive adenocarcinoma	>70%
HGCA 10	65/F	3.7	Hepatic flexure	Tubulovillous adenoma with high grade dysplasia	Invasive adenocarcinoma	>70%
HGCA 11	65/M	3.0	Sigmoid	Tubulovillous adenoma with high grade dysplasia	Invasive adenocarcinoma	>70%
HGCA 12	70/M	1.2	rectum	Tubulovillous adenoma with high grade dysplasia	No	>70%

### Whole-exome sequencing and copy number inference

WES was performed for genomic DNA from the HGCA and matched normal samples using Agilent SureSelect Human All Exome 50Mb kit (Agilent Technologies) and Illumina HiSeq2000 platform. The collection and processing of the sequencing data was performed as previously described [42]. The preparation of genomic DNA libraries and 101 bp paired-end sequencing reads was performed according to the manufacturer's instructions. Firstly, we used Burrows-Wheeler Alignment tool (BWA) to align the paired-end sequences onto the UCSC hg19 human reference genomes [43]. Local alignment and score recalibration of the sequencing data were performed, using the Genome Analysis ToolKit [44]. Additional downstream analyses, following alignment, were performed using Picard ( http://picard.sourceforge.net) and Samtools [45]. We used Mutect and SomaticIndelDetector to call somatic single nucleotide variants (SNVs) and small insertions/deletions (indels) by comparing the sequencing data from the adenoma samples with those from the matched normal tissue samples [44, 46]. ANNOVAR package was used for the analysis of mutations on coding sequences, and for the functional annotation of somatic variants [47]. For the identification of copy number alteration, we used VarScan 2 to obtain the read depth differences between the tumors and matched normal exome sequencing data [48]. The GC-corrected read depth was log_2_-transformed and segmented using circular binary segmentation algorithm [49].

### Validation of the whole-exome sequencing

We validated mutations of 12 genes either by digital-PCR or Sanger sequencing as described previously [50]. Digital-PCR was performed using the TaqMan Genotyping assay and the QuantStudio 3D digital PCR system (Life Technologies) according to the manufacturer's protocol.

## SUPPLEMENTARY MATERIALS FIGURES AND TABLES










