# Supplementary material for: Whole-exome sequencing identified mutational profiles of high-grade colon adenomas
Source: Oncotarget. 2016 Dec 25;8(4):6579–88. doi: 10.18632/oncotarget.14172 (PMC5351654; doi:10.18632/oncotarget.14172)
Supplement: Supplementary file 1 [file oncotarget-08-6579-s001.pdf]

# Whole-exome sequencing identified mutational profiles of high-grade colon adenomas

## SUPPLEMENTARY TABLES AND FIGURES

**Supplementary Table 1: The description of whole-exome sequencing data**

| Samples* | Sequencing reads | Mapped reads (%) | Mapped reads in exon (%) | Coverage (mean)** | % of bases (>= 20 reads)** |
|---|---|---|---|---|---|
| HGCA 1N | 71,790,807 | 70,664,617 (98.4%) | 49,550,187 (69.0%) | 85.0 | 94.4% |
| HGCA 1T | 81,856,114 | 80,842,075 (98.8%) | 60,763,865 (74.2%) | 104.1 | 95.9% |
| HGCA 2N | 76,800,262 | 75,839,271 (98.8%) | 56,058,266 (73.0%) | 95.6 | 95.0% |
| HGCA 2T | 76,583,998 | 75,572,118 (98.7%) | 54,199,481 (70.8%) | 92.5 | 94.9% |
| HGCA 3N | 78,350,099 | 77,375,208 (98.8%) | 57,096,416 (72.9%) | 97.7 | 94.9% |
| HGCA 3T | 74,600,212 | 73,699,784 (98.8%) | 53,073,976 (71.1%) | 90.8 | 94.5% |
| HGCA 4N | 75,254,402 | 74,300,376 (98.7%) | 53,486,317 (71.1%) | 91.0 | 95.0% |
| HGCA 4T | 79,017,342 | 78,027,767 (98.8%) | 56,450,408 (71.4%) | 96.7 | 95.2% |
| HGCA 5N | 77,129,411 | 76,205,180 (98.8%) | 55,259,347 (71.6%) | 94.2 | 95.3% |
| HGCA 5T | 78,482,455 | 77,560,553 (98.8%) | 56,649,509 (72.2%) | 96.4 | 95.5% |
| HGCA 6N | 77,334,827 | 76,419,755 (98.8%) | 55,115,105 (71.3%) | 94.3 | 94.7% |
| HGCA 6T | 82,322,361 | 81,478,814 (99.0%) | 59,839,243 (72.7%) | 101.9 | 95.5% |
| HGCA 7N | 91,167,035 | 90,417,108 (99.2%) | 66,116,912 (72.5%) | 112.8 | 96.3% |
| HGCA 7T | 88,752,925 | 87,726,673 (98.8%) | 60,046,191 (67.7%) | 102.9 | 95.8% |
| HGCA 8N | 126,637,990 | 125,869,988 (99.4%) | 85,676,904 (72.4%) | 149.7 | 96.9% |
| HGCA 8T | 116,285,070 | 115,536,400 (99.4%) | 78,530,086 (68.0%) | 136.7 | 96.6% |
| HGCA 9N | 130,274,028 | 129,023,866 (99.0%) | 78,725,426 (64.8%) | 136.8 | 96.6% |
| HGCA 9T | 126,229,238 | 125,485,582 (99.4%) | 86,338,226 (72.9%) | 150.6 | 97.1% |
| HGCA 10N | 120,764,798 | 120,110,578 (99.5%) | 83,285,267 (73.4%) | 145.8 | 97.2% |
| HGCA 10T | 111,099,170 | 110,502,460 (99.5%) | 75,574,953 (71.2%) | 131.1 | 96.5% |
| HGCA 11N | 106,510,816 | 86,796,706 (81.5%) | 18,541,263 (31.0%) | 30.1 | 57.3% |
| HGCA 11T | 111,327,540 | 110,697,002 (99.4%) | 78,987,996 (74.9%) | 137.8 | 96.7% |
| HGCA 12N | 108,292,690 | 91,468,110 (84.5%) | 26,500,556 (28.0%) | 43.1 | 76.4% |
| HGCA 12T | 113,953,908 | 113,662,200 (99.7%) | 85,745,276 (80.0%) | 149.2 | 97.1% |

*The tumor and matched normal genomes are discriminated with the use of 'T' and 'N', respectively.
**The mean coverage and the % of bases (>= 20 reads) were calculated onto the targeted regions (Agilent SureSelect 50Mb exon).

**Supplementary Table 2: A list of somatic mutations identified in 12 high-grade colon adenoma samples by exome sequencing (XLS).**

See Supplementary File 1

**Supplementary Table 3: Non-silent somatic mutations identified in 12 high-grade colon adenoma samples (XLS).**

See Supplementary File 2

**Supplementary Table 4: Copy number alterations identified across 12 colorectal adenoma samples (XLS).**

See Supplementary File 3

**Supplementary Table 5: Functional annotation and Pathway analysis of somatic mutations using DAVID (XLS).**

See Supplementary File 4

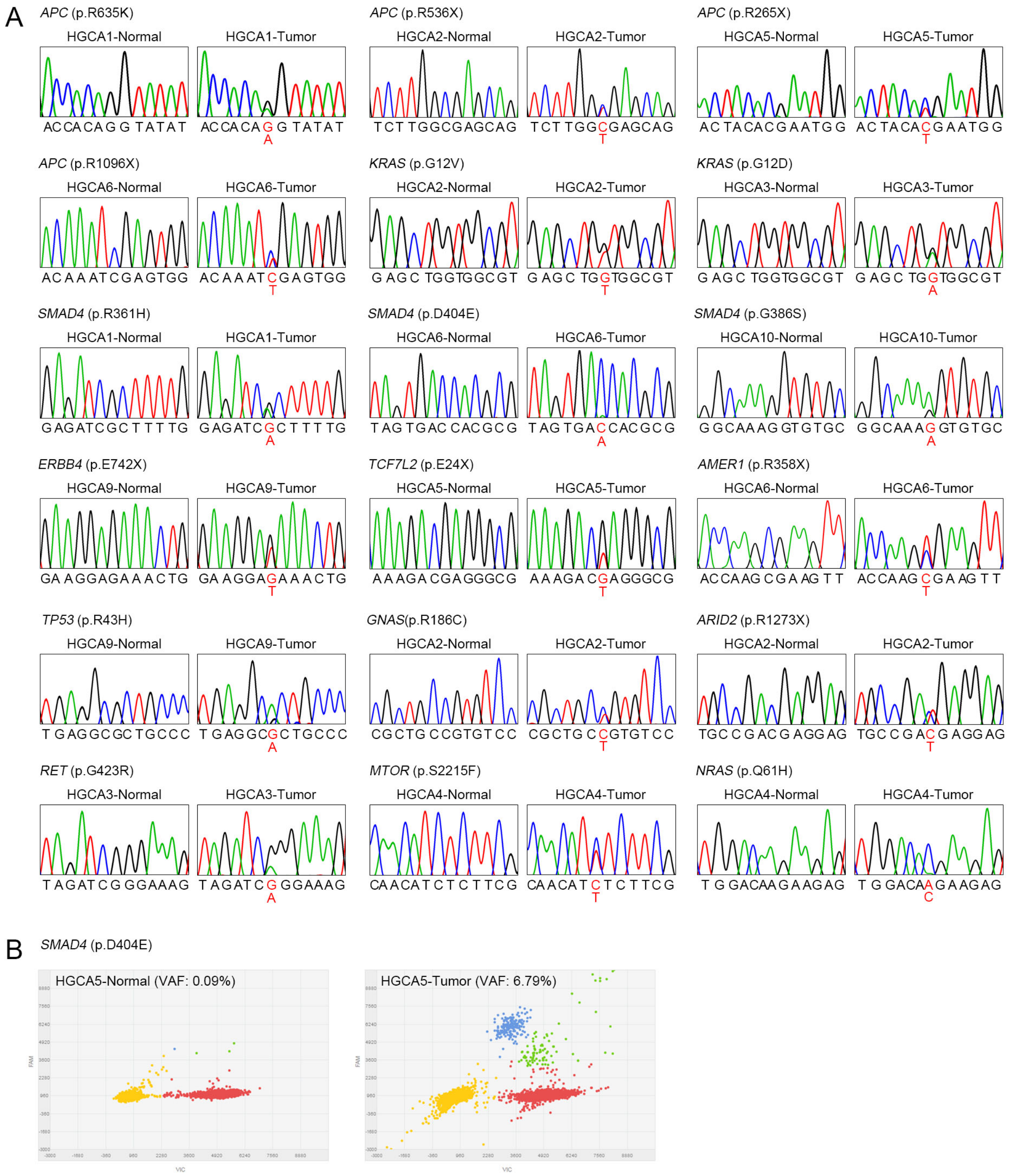


**Supplementary Figure 1: Validation of mutations identified in the high-grade colon adenoma samples by either Sanger sequencing A. or digital PCR B.**

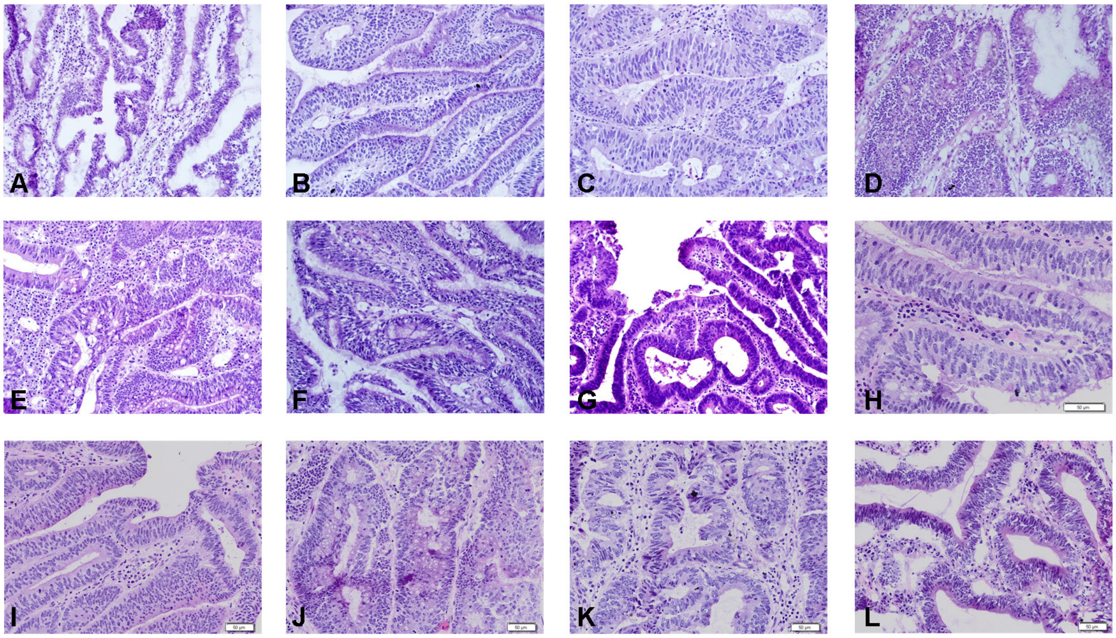

**Supplementary Figure 2: Representative figures of the high-grade colon adenoma cases (x200) A. HGCA 1T, B. HGCA 2T, C. HGCA 3T, D. HGCA 4T, E. HGCA 5T, F. HGCA 6T, G. HGCA 7T, H. HGCA 8T, I. HGCA 9T, J. HGCA 10T, K. HGCA 11T, L. HGCA 12T**
